# Analysis of Mutations in 7 Genes Associated with Neuronal Excitability and Synaptic Transmission in a Cohort of Children with Non-Syndromic Infantile Epileptic Encephalopathy

**DOI:** 10.1371/journal.pone.0126446

**Published:** 2015-05-07

**Authors:** Anna Ka-Yee Kwong, Alvin Chi-Chung Ho, Cheuk-Wing Fung, Virginia Chun-Nei Wong

**Affiliations:** Division of Paediatric Neurology / Developmental Behavioural Paediatrics / Neurohabilitation, Department of Paediatrics and Adolescent Medicine, Li Ka Shing Faculty of Medicine, The University of Hong Kong, Hong Kong SAR, China; Charité Universitätsmedizin Berlin, NeuroCure Clinical Research Center, GERMANY

## Abstract

Epileptic Encephalopathy (EE) is a heterogeneous condition in which cognitive, sensory and/or motor functions deteriorate as a consequence of epileptic activity, which consists of frequent seizures and/or major interictal paroxysmal activity. There are various causes of EE and they may occur at any age in early childhood. Genetic mutations have been identified to contribute to an increasing number of children with early onset EE which had been previously considered as cryptogenic. We identified 26 patients with Infantile Epileptic Encephalopathy (IEE) of unknown etiology despite extensive workup and without any specific epilepsy syndromic phenotypes. We performed genetic analysis on a panel of 7 genes (*ARX*, *CDKL5*, *KCNQ2*, *PCDH19*, *SCN1A*, *SCN2A*, *STXBP1*) and identified 10 point mutations [*ARX* (1), *CDKL5* (3), *KCNQ2* (2), *PCDH19* (1), *SCN1A* (1), *STXBP1* (2)] as well as one microdeletion involving both *SCN1A* and *SCN2A*. The high rate (42%) of mutations suggested that genetic testing of this IEE panel of genes is recommended for cryptogenic IEE with no etiology identified. These 7 genes are associated with channelopathies or synaptic transmission and we recommend early genetic testing if possible to guide the treatment strategy.

## Introduction

Epileptic Encephalopathy (EE) is a heterogeneous condition in which cognitive, sensory and/or motor functions deteriorate as a consequence of epileptic activity, which consists of frequent seizures and/or major interictal paroxysmal activity [1]. This concept was formally recognized in 2001 and subsequent International League Against Epilepsy (ILAE) reports. The 2010 report of the ILAE Commission on Classification and Terminology stated that “Epileptic Encephalopathy embodies the notion that the epileptic activity itself may contribute to severe cognitive and behavioral impairments above and beyond what might be expected from the underlying pathology alone. These impairments may be global or more selective and they may occur along a spectrum of severity [2]”. EE may occur at any age, but the phenomenon is most common and severe in infancy and early childhood, which is the most critical period of brain maturation [3]. However, many neonates or infants with EE do not fit into any of the proposed epileptic syndromes [4].

Etiologies for EE can be due to 1) congenital structural brain abnormalities, 2) metabolic diseases, 3) recognizable dysmorphic syndromes, 4) non-syndromic monogeneic causes, or 5) environmental causes [5]. Structural brain abnormalities, either congenital (such as cortical malformations) or acquired (such as hypoxic ischemic insults), are the most common cause of early onset EE [4]. However, in approximately one third of patients with EE, the underlying cause remains unknown after extensive investigations [6].

With the advancement of gene diagnostics technology, genetic defects have been increasingly recognized as causes of early onset EEs previously considered cryptogenic. Genes including *ARX*, *CDKL5*, *KCNQ2*, *PCDH19*, *SCN1A*, *SCN2A* and *STXBP1* involved in ion channelopathies, neuronal transmission, brain development or synaptic functions were reported to be associated with EEs. [7]. Previously, these genes were only found to be associated with specific epilepsy syndromes, but recent reports widened the clinical spectrum of these gene-associated disorders. For example, *KCNQ2* mutations have been reported in benign familial neonatal seizures (BFNS) and in severe neonatal EE (NEE) with quadriplegia and Ohtahara syndrome [8–12]. *STXBP1* mutations were first reported in patients with Ohtahara syndrome [13] and later identified in patients with infantile spasm, DS, West syndrome and nonsyndromic early onset EE [14–16] and quadriplegia [13].

As the clinical phenotype for these gene-associated EEs was expanding and overlapping, there was an anticipated difficulty in selecting only one candidate gene for molecular evaluation in patients with EE who did not fit into any epilepsy syndrome classified according to ILAE. We had therefore selected 7 genes (*ARX*, *CDKL5*, *KCNQ2*, *PCDH19*, *SCN1A*, *SCN2A*, and *STXBP1*) for screening our patients with non-syndromic Infantile Epileptic Encephalopathies (IEE) after extensive metabolic and neuroimaging workup had been negative. These genes have been associated for years with a fairly well-established spectrum of clinical phenotypes. They play a critical role in neurotransmission and synaptic function, which could be an important mechanism of IEE. Identification of an underlying genetic cause is essential to provide information on prognosis and avoid unnecessary investigations. Moreover, the possibility of prenatal diagnosis and selection of appropriate anticonvulsants might follow.

## Methods

### Ethics Statement

This study was approved by the Institutional Review Board of the Hong Kong West Cluster and the University of Hong Kong (IRB Ref. No.: UW 11–190). Written consent was obtained from the parents of our patients.

### Patient samples and clinical diagnosis

The study was conducted in Queen Mary Hospital and Duchess of Kent Children Hospital, two affiliated hospitals of The University of Hong Kong. We included patients who satisfied the ILAE definition of EE and with seizure onset before 24 months of age. According to the ILAE Commission on Classification and Terminology, infants are referred to those less than one year of age [2]. We had included children with seizure onset before the age of two years in order to capture those with late IEE [17] or IEE with late onset spasms [18]. We excluded patients with definite evidence of brain insult, confirmed disorder of cortical development by magnetic resonance imaging, neurocutaneous disorders, syndromal disorders and confirmed or highly suspected neurometabolic disorders based on clinical (multi-system involvement including organomegaly or skeletal changes) and biochemical markers. Extensive neurometabolic evaluations conducted for all these patients were negative (blood for amino acid, biotinidase, ammonia, lactate, glucose, very long chain fatty acids including phytanic and pristanic acids, transferrin isoform electrophoresis, total homocysteine, copper, coeruloplasmin, creatine and guanadinoacetate; urine for purine and pyrimidine screening, creatine, guanadinoacetate and organic acid; cerebrospinal fluid for glucose, lactate, protein, amino acid, neurotransmitters and 5-methyltetrahydrofolate). All patients failed to show any positive response to a trial of intravenous pyridoxine up to 300 mg under electroencephalography monitoring, followed by adequate trials of oral pyridoxine, pyridoxal phosphate and folinic acid [19]. We also excluded patients who fit into distinct electroclinical syndromes proposed by the ILAE and those not actively followed up in our centre.

Data variables collected from the medical charts included demographic information (gender, ethnicity, age at seizure onset and latest follow up), family history (febrile convulsion, epilepsy, intellectual disability and other neurological diseases), epilepsy details (seizure types at onset and latest follow up, seizure frequency and evolution, history of status epilepticus, anti-epileptic medications used), neurological examination findings (upper motor neuron syndrome, hypotonia, movement disorders [dystonia, choreoathetosis, myoclonus, ataxia, parkinsonism], microcephaly, macrocephaly, dysmorphism), investigations (MRI brain and EEG results), mortality and other associated clinical features (autism spectrum disorder and other neurobehavioral disorders such as attention deficit hyperactivity disorder, visual impairment, hearing impairment, ability of independent walking and oromotor dysfunction requiring nasogastric tube or gastrostomy feeding). Information regarding the developmental status at the time of seizure onset and latest follow up was collected as well. Either formal neuropsychological testing (using Griffiths Mental Developmental Scale or HK-WISC) or best clinical assessment (based on developmental milestones recorded in the medical charts) were used to classify development or intelligence as normal, mildly delayed, moderately delayed or severely delayed.

All patients were screened for mutations of 6 genes (*ARX*, *CDKL5*, *KCNQ2*, *SCN1A*, *SCN2A* and *STXBP1*). Mutation analysis of *PCDH19* was only performed in female patients as the *PCDH19*-associated X-linked IEE mainly affect female with heterozygous mutations.

### Point mutation analysis

Genomic DNA samples of the patients were extracted from peripheral blood using Flexigene DNA Kit (Qiagen GmbH, Germany). All exons covering the coding regions as well as the splice junctions were amplified by polymerase chain reaction (PCR) using oligonucleotide primers designed based on the reference genomic sequence (Table 1) of different genes. PCR contained 0.1 μg of genomic DNA as template, 5 pmol of each primer, 200 μM of deoxyribonucleoside triphosphates, and 0.5 U HotStarTaq Plus DNA polymerase (Qiagen) in 1X Qiagen PCR buffer. PCR was carried out with initial enzyme activation at 95°C for 5 minutes, followed by 50 cycles of denaturation at 94°C for 30 seconds, annealing at 60°C for 1 minute and extension at 72°C for 1.5 minutes, with a final extension at 72°C for 10 minutes. For those templates with high degree of secondary structures or high GC-contents, 1x Q-Solution (Qiagen) was included in the PCR mixtures. If the non-specific products could not be eliminated by adding Q-Solution, a higher initial activating temperature of 98°C and denaturing temperature of 96°C were used (Table 1). The quality and quantity of PCR products were checked by agarose gel electrophoresis. PCR products were directly used for sequencing reaction by Bigdye Terminator v3.1 Cycle Sequencing Kit (Applied Biosystem, Foster City, CA) and analyzed on a 3730xl DNA analyzer (Applied Biosystems).

**Table 1:** GenBank accession numbers, chromosome positions and PCR conditions for exon amplifications of the 7 IEE genes.

Gene	GenBank accession no.		Chromosome position	Coding exons	PCR conditions
	Gene	mRNA			
***ARX***	NG_008281	NM_139058	Xp21.3	Exon 1, 3–5	Higher temperature
				Exon 2	Higher temperature and Q-solution added
***CDKL5***	NG_008475	NM_003159	Xp22	Exon 2–21	Default
***KCNQ2***	NG_009004	NM_172107	20q13.3	Exon 1, 6–7	Q-solution added
				Exon 2–5, 8–16	Default
				Exon 17	Higher temperature and Q-solution added
***PCDH19***	NG_021319	NM_001184880	Xq22	Exon 1–6	Default
***SCN1A***	NG_011906	NM_001165963	2q24.3	Exon 1–26	Default
***SCN2A***	NG_008143	NM_001040142	2q24.3	Exon 1–29	Default
***STXBP1***	NG_016623	NM_003165	9q34.1	Exon 1	Q-solution added
				Exon 2–20	Default

Homology analyses with the reference genomic sequence (Table 1) were performed using NCBI program BLAST. The numbering for each mutation was taken from the start codon with +1 corresponding to the A of the ATG in the reference sequence (Table 1). Mutations were discriminated from single nucleotide polymorphisms (SNP) with allele frequency > 0.01 reported in NCBI SNP and Ensembl SNP database. The parental DNA was collected and sequenced to distinguish between *de novo* and familial variants.

### Pathogenicity assessment of the mutations

Evolutionary conservation analysis was performed to predict whether the amino acid substitution in missense mutations would affect protein function based on the degree of conservation at the affected residues. Besides, online sequence homology-based tool, Sorting Intolerant from Tolerant (SIFT), Polymorphism Phenotyping v2 (PolyPhen-2) and Align-Grantham variation/Grantham deviation (Align-GVGD) analysis have been used to predict whether the mutation would interfere with the protein function. We have described them previously [20]. The two splice site mutations in intron regions were analyzed by another online software tool, the Automated Splice Site Analyses (Laboratory of Human Molecular Genetics and Genomic Disorders, UWO, CA, https://splice.uwo.ca/) [21].

### Multiplex ligation-dependent probe amplification (MLPA)

For identification of copy number variations (CNVs) of the intragenic regions or entire genes, MLPA was used. It made use of a single PCR primer pair for all the probes to determine the copy number of all sequences in a single multiplex PCR based reaction followed by capillary electrophoresis.

For the *ARX*, *CDKL5*, *KCNQ2*, *PCDH19* and *SCN1A* genes, commercial MLPA probemixes (SALSA P189 *CDKL5* probemix for both *CDKL5* and *ARX*, SALSA P166 *KCNQ2* probemix for *KCNQ2* and SALSA P137 *SCN1A* probemix for *SCN1A*, MRC Holland, Amsterdam, The Netherlands) as well as the SALSA MLPA reagent kit (MRC Holland) were used. For *SCN2A* and *STXBP1*, commercial MLPA kits were not available and synthetic MLPA probes were designed by the online software H-MAPD suggested by the protocol (http://bioinform.arcan.stonybrook.edu/mlpa2/cgi-bin/mlpa.cgi) and synthesized for all the exons according to the guidelines and protocol provided by MRC Holland. The MLPA procedures were performed according to the manufacturer’s protocols. Fragment analysis of PCR products was performed on the ABI 3130xl capillary sequencer (Thermo Fisher Scientific, Waltham, MA) by using GeneScan TM-500LIZ as size standards (Thermo Fisher Scientific) and HiDi formamide (Thermo Fisher Scientific). The GeneScan results were analyzed using Coffalyser software (MRC Holland). The peak area of a given exon was divided by the sum of 12 reference peak areas for each individual sample. The final ratio was obtained by dividing this relative peak area of the corresponding exon by the averaged normal control peak area. Thresholds of <0.65 were set for deletions and >1.35 for duplications.

## Results

### Clinical characteristics (Table 2)

Twenty-six IEE patients, whose etiology was unknown and who did not fit into specific electroclinical syndromes, were identified from our registry. The majority (24/26, 92%) of patients were from Asia. Only 2 patients had an ethnic origin other than Asian. One was African-French (patient 32). Another was Portuguese-Chinese (patient 44). The majority (21/26, 81%) of the selected IEE cohort were pure Chinese.

**Table 2:** Clinical characteristics of 26 patients with infantile epileptic encephalopathies of unknown etiology.

Case no.	Ethnic origin	Sex/ Age	Mutation	Age of first seizure (months)	Type of seizure at onset*	Seizure types developed*	Number of regular anti-epileptic drugs	Seizure evolution	Development at most recent follow up	Associated clinical features
2	Chinese	M/3	*STXBP1* frameshift	0.5	S	G, M, S	5	>50% reduction	Severe delay	UMN signs; dystonia; microcephaly; CVI; laryngomalacia; failure to thrive
9	Chinese	F/6	*ARX* nonsense	1	F	A, At, F, G, M	4	>25% but <50% reduction	Severe delay	UMN signs; dystonia; microcephaly; CVI
15	Chinese	F/9	*CDKL5* missense	3	F	F	6	>50% reduction	Severe delay	Autism
25	Philippine	F/12	*CDKL5* nonsense	3	S	G, M, S	10	>50% reduction	Severe delay	Dystonia; hand stereotypies
28	Chinese	F/4	*KCNQ2* indels	<0.25	F	F, G, S, O (breath-holding attacks)	7	>50% reduction	Severe delay	UMN signs; dystonia
30	Chinese	M/14	*KCNQ2* splice site	0.25	G	F, G	2	Seizure free	Mild delay	Nil
32	African- French	F/6	*STXBP1* splice site	0.5	F	F, G, S	7	>50% reduction	Severe delay; developmental regression	Dystonia; CVI
33	Chinese	F/18	*PCDH19* frameshift	7	F, G	F, G	4	Seizure free	Mild delay	Behavioral problem
36	Chinese	F/3	*CDKL5* frameshift	10	G	G, S	8	>25 but <50% reduction	Severe delay	Nil
40	Chinese	F/16	*SCN1A* splice site	6	F	F, G	10	>50% reduction	Severe delay	Nil
47	Chinese	M/5	*SCN1A* & *SCN2A* whole gene deletion	2	F	F, S, G, M	7	>50% reduction	Severe delay; developmental regression	Microcephaly
1	Chinese	F/9	Nil	6	At, M	A, At, F, G, M, S	6	>25% but <50% reduction	Severe delay; developmental regression	UMN signs
7	Chinese	M/4	Nil	12	F	F, G, M, O (hypomotor seizures)	3	>25% but <50% reduction	Moderate delay	Microcephaly
8	Chinese	F/15	Nil	7	S	A, G, M, S	11	Static	Severe delay; developmental regression	Autism; features of Rett syndrome
12	Chinese	M/3	Nil	3	S	M, S	6	Static	Severe delay	Left foot dystonia; microcephaly
13	Chinese	M/6	Nil	6	G	A, F, G, M	7	>50% reduction	Severe delay; developmental regression	Microcephaly
18	Indian	M/8	Nil	2	S	G, M, S	6	>50% reduction	Severe delay	UMN signs; Dystonia; CVI
20	Chinese	M/4	Nil	6	S	S	8	Static	Severe delay	UMN signs; dystonia; microcephaly; oromotor dysfunction required gastrostomy
21	Chinese	F/15	Nil	8	S	G, S	10	>50% reduction	Mild delay; developmental regression	Autism; ADHD
23	Chinese	F/2	Nil	4	G, M, S	G, M, S	4	>50% reduction	Severe delay	Dystonia; CVI
24	Chinese	M/3	Nil	0.25	S	A, F, G, M, S	2	>50% reduction	Severe delay	UMN signs; dystonia; microcephaly
31	Singaporean- Chinese and Malaysian- Chinese	F/7	Nil	0.25	F	F, G	7	>50% reduction	Severe delay	Autism
34	Chinese	M/8m	Nil	4	F	F, G	7	Static	Severe delay; developmental regression	CVI
41	Chinese	M/9	Nil	<0.25	F	F, S	5	>25 but <50% reduction	Mild delay	Autism
44	Portuguese- Chinese	M/3	Nil	14	At, G	At, G	3	Static	Moderate delay	Nil
45	Chinese	M/9	Nil	1	S	F, G, S	2	>50% reduction	Normal	Nil

*S: spasm; M: myoclonic seizure; F: focal seizure with or without generalization; G: generalized tonic / clonic / tonic-clonic seizure; A: absence seizure; At: atonic seizure; O: other seizure type.

The mean age of seizure onset was 4.1 months (SD 3.9 months), while the median was 3 months. Epileptic spasm was the most common type of seizure at onset. 10 out of 26 (38%) had epileptic spasm as the first seizure type. Most of the children (92%) developed multiple types of seizures with time. Concerning the seizure evolution, 16 patients (62%) had more than 50% seizure reduction and 5 patients (18%) had 25–50% seizure reduction. Concerning neurodevelopment, 19 patients (73%) were severely developmentally delayed at the latest evaluation. Seven patients (27%) had developmental regression, which was considered to be the hallmark of EE and was defined as loss of acquired skills. This was compatible with EE which has the tendency to abate, discontinue or even stop, but often with serious neurocognitive deficits [22]. It was worth noting that 10 patients (38%) had a movement disorder exclusively in the form of dystonia, either generalized or focal and 7 patients (27%) had an upper motor neuron syndrome.

### Mutation analysis

**Fig 1:**
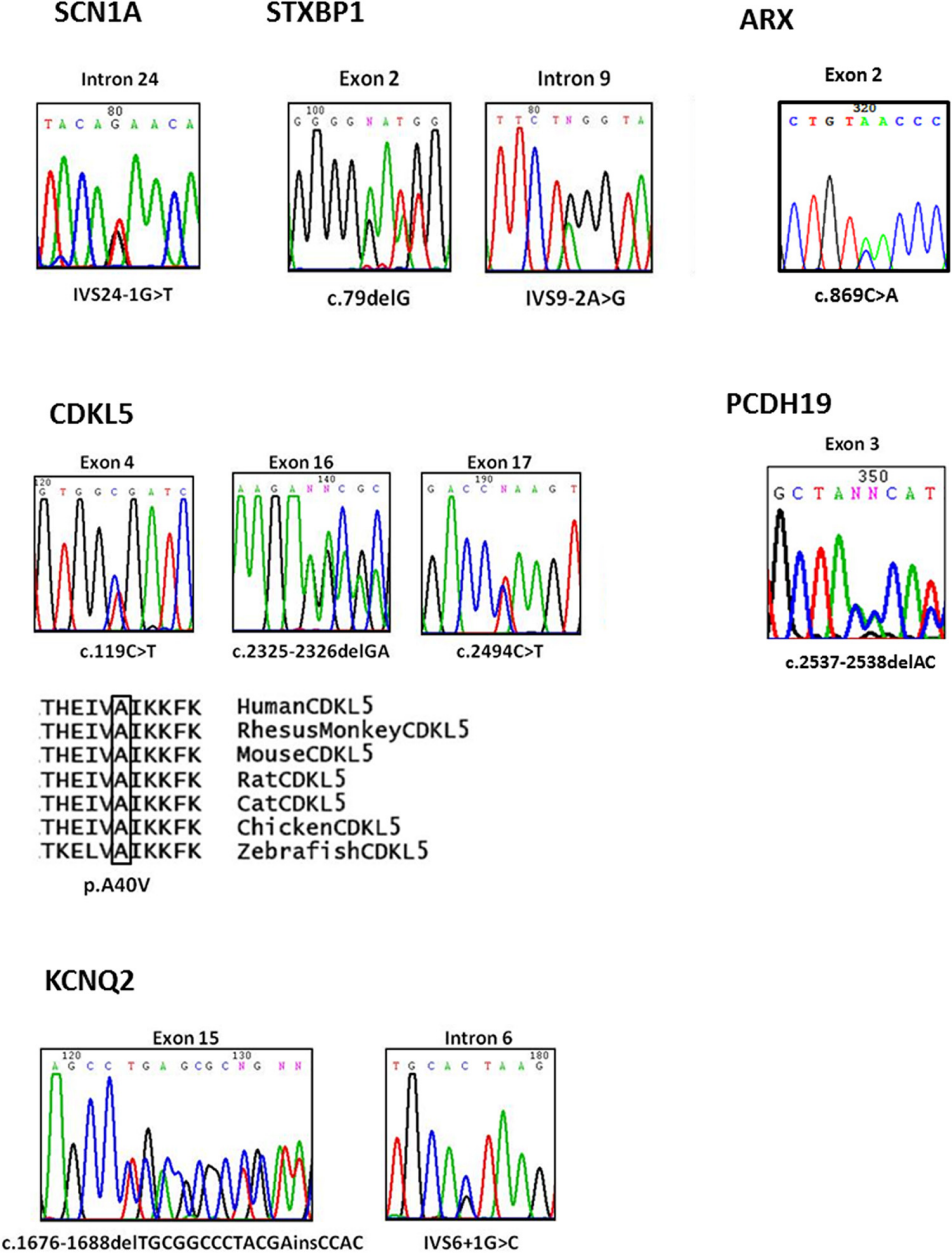
Mutations of the *ARX*, *CDKL5*, *KCNQ2*, *PCDH19*, *SCN1A*, *SCN2A* and *STXBP1* genes found in the 10 patients and evolutionary conservation analyses for the missense mutation.

Eleven out of 26 patients (42%) were found to have mutations among the 7 genes (Fig 1 and Table 2). All mutation details have been summarized in Table 3. Most of the variants were identified to be truncating and only one of them (p.A40V) was a missense mutation. Evolutionary conservation analysis showed that the affected amino acid residue of this missense mutation was highly conserved (Fig 1). SIFT, Polyphen-2 and Align-GVGD (Grantham variation: 0; Grantham deviation: 65.28; Class 65) analyses predicted that the missense mutation is probably damaging. Nine of the mutations were novel and the 2 *CDKL5* mutations have been reported previously [23–26]. The three splice site mutations, IVS24-1G>T, IVS9-2A>G and IVS6+1G>C, were predicted to form a leaky acceptor splice site, abolish the acceptor or donor splice site respectively. There were no microdeletions or CNVs for *ARX*, *CDKL5*, *KCNQ2*, *PCDH19* and *STXBP1* by MLPA analysis.

**Table 3:** Variants of the *ARX*, *CDKL5*, *KCNQ2*, *PCDH19*, *SCN1A*, *SCN2A* and *STXBP1* genes found in the 11 IEE patients.

Gene	patient	Type of mutation	Exon	Mutation		Location in protein	Inherited/ *De novo*	Reported/Novel
				Nucleotide	Amino acid			
***ARX***	9	Heterozygous nonsense	2	c.869C>A	p.S290X	Upstream of both paired- homeodomain and aristaless domain	*De novo*	Novel
***CDKL5***	15	Heterozygous missense	4	c.119C>T	p.A40V	Located at the ATP-binding site	Parents’ DNA not available	Reported [23–26]
	25	Heterozygous nonsense	17	c.2494C>T	p.Q832X	Upstream of two putative sites at C-terminal	*De novo*	Novel
	36	Heterozygous deletion leading to frameshift	16	c.2325-2326delGA	p.K776fsX799	Upstream of three putative sites at C-terminal	Parents’ DNA not available	Reported [23]
***KCNQ2***	28	Heterozygous deletion and insertion (indels)	15	c.1676-1688delTGCGGCCCTACGAinsCCAC	p.L559-D563delinsPT	Highly conserved assembly domain (A domain) in C-terminal	*De novo*	Novel
	30	Heterozygous splice site	Intron 6	IVS6+1G>C	-	Junction between exon 6 &7 is located at transmembrane domain 6	*De novo*	Novel
***PCDH19***	33	Heterozygous deletion leading to frameshift	3	c.2537-2538delAC	p.N846fsX861	Located at cytoplasmic domain before CM1 and CM2 motifs	Parents’ DNA not available	Novel
***SCN1A***	40	Heterozygous splice site	Intron 24	IVS24-1G>T	-	Junction between domain 3 & 4	Only mother DNA available with no mutation	Novel
***SCN1A +SCN2A***	47	One copy deletion of the entire *SCN1A* and *SCN2A* genes	all	-	-	-	*De novo*	Novel
***STXBP1***	2	Heterozygous deletion leading to frameshift	2	c.79delG	p.E27fsX36	Domain 1	*De novo*	Novel
	32	Heterozygous splice site	Intron 9	IVS9-2A>G	-	Domain 3a	Parents’ DNA not available	Novel

## Discussion

### High occurrence of mutations in our selected IEE gene panel

We had attempted to apply the panel approach of genetic testing on subjects with IEE. These seven genes were reported to be involved in IEE. We found that the yield of the current gene panel analysis on IEE patients with unknown etiology was up to 42%. This is also the first study on a cohort of mainly (81%) Chinese patients. Upon review of the clinical characteristics of our patients with a positive mutation, no single or a group of clinical features could assist in pinpointing a particular candidate gene for analysis. We found that of 10 patients (38%) with dystonia, 5 were positive for one of the 5 gene mutations (*ARX*, *CDKL5*, *KCNQ2*, *SCN2A and STXBP1*) and dystonia had not been highlighted as a key clinical feature for selecting genetic mutations for IEE. As for those patients with mutations in the same gene, dystonia was not necessarily present during the clinical evolution [25% (1 out of 4) in *CDKL5*; 50% (1 out of 2) in *KCNQ2*; 100% (1 out of 1) in *SCN2A*, (1 out of 1) in *ARX* and (2 out of 2) in *STXBP1*].

### Mutation in *SCN1A*, *SCN2A* & *KCNQ2* for neuronal excitability

Ion channelopathies play a prominent role in the development of IEE. To date, more than 600 variants of *SCN1A* encoding for a voltage-gated sodium channel have been identified and most mutations were found in patients with DS [27]. Our previous study identified more than 70% of *SCN1A* mutations in a group of Chinese children with DS [20]. In contrast to *SCN1A*, much fewer *SCN2A* mutations were reported in previous literatures. Mutations were recently identified in severe EE including DS, infantile spasm and Ohtahara syndrome [28–31]. Our group also identified a SCN2A mutation in a patient with infantile spasm and severe intellectual disability previously [32]. In the present study, one *SCN1A* mutation and one microdeletion involving both *SCN1A* and *SCN2A* were identified. They were deleterious to the protein and the abnormal sodium channel function leading to severe phenotypes could result from haploinsufficiency as suggested previously [33, 34].

Mutations of *KCNQ2* encoding the voltage-gated potassium channels were identified in patients with neonatal EE [8]. *KCNQ2* is expressed in broad regions of the brain and the gene products form heteromultimeric channels that mediate the M-current that inhibit the neuronal excitability [35]. Two IEE patients in the present study were found to have *KCNQ2* mutations. The first mutation was a deletion-insertion mutation that replaced a short fragment LRPYD by two amino acids (PT) in the protein. The short fragment (LRPYD) is located in a highly conserved domain (A-domain) of C-terminal of KCNQ2 necessary for subunit interactions to form homo- or heteromeric channels to reach the surface [36, 37]. Another *KCNQ2* mutation was a splice site mutation which may cause aberrant splicing and disrupt the protein at the position within transmembrane domain 6.

### Mutation in *PCDH19* & *STXBP1* for synaptic transmission

*PCDH19* belongs to the PCDHδ2 subgroup of PCDH family consisting of 6 extracellular cadherin (EC) repeats. It is involved in calcium-dependent cell-cell adhesion at the synaptic membrane [38, 39] and it was hypothesized that the cellular interference was the main pathogenic mechanism associated with *PCDH19* mutations [40]. Previously, we have identified *PCDH19* mutations in two of our patients [20]. In the present study, a *PCDH19* frameshift mutation (p.N846fsX861) was identified. This mutation terminates the protein at the cytoplasmic domain and abolishes the conserved CM1 and CM2 motifs [41, 42]. Wolverton & Lalande [42] suggested that CM2 may play a functional role for mediating intracellular signal transduction.

*STXBP1* encoding for the neural-specific syntaxin-binding protein has long been discovered for regulation docking and fusion of synaptic vesicles through interaction with syntaxin in the SNARE complex for neurotransmitter release [43, 44]. Until recently, *STXBP1* mutations were identified to be associated with different forms of early-onset EE including Ohtahara syndrome, West syndrome and infantile spasms [7, 13–15]. STXBP1 is a horse-shoe shaped protein with 3 domains while domain 1 and 3a form the central cavity providing the binding surface for syntaxin [45]. In the present study, c.79delG is a novel frameshift mutation forming a stop codon in the early reading frame and IVS9-2A>G is a novel splice site mutation that may possibly disrupt the protein at domain 3a necessary for syntaxin binding.

### Role of *CDKL5* and *ARX* mutation in synaptic development

The CDKL5 protein belongs to the family of serine/threonine kinases which is characterized by an N-terminal catalytic domain [46]. In the past ten years, *CDKL5* mutations were found to be associated with early-onset EE. In the present study, a relatively high percentage of *CDKL5* mutations (14%) was found in non-syndromic IEE patients. p.A40V found in the present study is one of the mutation hot-spots located at the highly conserved ATP-binding site (amino acid 14–47) of the catalytic domain reported previously in 4 different studies including 5 patients [23–26].

Although mutation hot-spots were found in the catalytic domain, many pathological alterations can still be found in the C-terminal region [46]. The frameshift (p.K776fsX799) and nonsense (p.Q832X) mutations identified in the present study may cause truncation of the C-terminus. The 2 truncating mutations located upstream from 2 and 3 putative sites which are essential for the cellular localization of the protein [47]. Evidence was provided previously that the C-terminus of CDKL5 is a negative regulator of catalytic activity of CDKL5 and required for a proper subnuclear localization by protein-protein interactions [47–49]. p.K776fsX799 has been reported previously and immunofluorescence data of the same study demonstrated that the truncated protein mislocalized to the cytoplasm [23]. The important role of CDKL5 for proper brain function and development elucidate the relationship of *CDKL5* mutations with neurodevelopmental disorders.

*ARX* encodes an important transcription factor that plays a significant role in the neuronal development of the brain [50]. In the present study, a heterozygous *ARX* mutation has been found in a female IEE patient with multiple seizure types, spastic dystonic quadriplegia and severe developmental delay. Although most affected females with *ARX* mutations showed relatively mild clinical outcomes as compared to males, severe cases were reported previously with various outcomes [51, 52]. These cases may have occurred due to skewed X-inactivation or post-zygotic mosaicism [52]. Further studies will be performed to illustrate the pattern of X-inactivation in the patient. The previous literature reported *ARX* mutation associated with IEE in female only rarely and only 2 cases with truncating *ARX* mutations have been reported previously [51, 53].

### Association of the seven IEE genes with synaptic transmission

In the present study, mutations were found in the genes involved in neuronal excitability (*KCNQ2*, *SCN1A*, and *SCN2A*), synaptic transmission (*PCDH19*, *STXBP1*) and synapse development (*ARX*, *CDKL5*). The study of relationship between neurotransmitter release and ion channels illustrated that impairment in structure and function of ion channels can actually modulate the synaptic transmission by changing the synaptic terminal excitability [54, 55]. The genetic defects found in the 7 genes may contribute directly or indirectly to the malfunction of synaptic transmission that may be an important mechanism for IEE. A recent comprehensive exome-sequencing study suggested that dysregulation of synaptic transmission plays an important role in the pathogenesis of EE as they demonstrated a significant enrichment of *de novo* mutations in genes annotated to be involved in synaptic transmission by pathway analysis [56].

### Recommended diagnostic flow for patients with IEE

Based on the findings in our study, we propose a diagnostic algorithm for patients with IEE. Through clinical history taking, physical examination and neuroimaging (magnetic resonance imaging of the brain), relatively straightforward etiologies can be identified. If an underlying etiology is still unknown, a detailed neurometabolic evaluation should be performed especially aiming for potentially treatable causes such as vitamin-responsive epilepsies. This should involve an adequate trial (dosage and duration) of pyridoxine, pyridoxal phosphate and folinic acid. For those patients still without an underlying cause found, molecular workup is recommended. Candidate gene(s) testing can be performed according to the recommendation by Ottman et al [57] if a patient fits into a certain electroclinical syndrome. Otherwise, depending on the availability of resources, mutation analysis of our selected panel of genes (*ARX*, *CDKL5*, *KCNQ2*, *PCDH19*, *SCN1A*, *SCN2A* and *STXBP1*) is an option which can have a yield of up to 42%. Sanger sequencing of the selected gene panel is a relatively simple and direct method which do not require various steps of library preparation and target capturing, platforms for next generation sequencing, bioinformatics and various filtering strategies. Besides, the problem of uneven coverage is one of the issues that have to be overcome in next generation sequencing. Sanger sequencing of the selected gene panel will be a good choice for small-scale mutational studies with fewer resources available. However, for the remaining patients without any positive yield, next generation sequencing is still the choice for identification of other causative genes.

In the present study, except for the finding of the whole gene deletion of *SCN1A* and *SCN2A* in one patient, all of the MLPA analysis showed negative results for the other 5 genes. Besides, negative MLPA results have been found for other putative IEE-associated genes including *NRXN1*, *GRIN2A* and *GRIN2B* in our study (in preparation). As the yield of genetic defects identified by MLPA was low, we do not suggest trying to identify copy number variations of IEE-associated genes by MLPA if the resources are limited.

### Conclusion

This study highlights that patients with non-syndromal IEE might not have specific phenotypes to guide candidate gene(s) selection. The yield of mutation analysis of seven selected genes of the IEE panel in this group of patients was 42%. Panel approach of genetic testing can be useful in investigating the underlying cause of IEE that do not fit into any distinct electroclinical syndromes and without any obvious etiologies including neurometabolic diseases.
